# Effects of combined training on metabolic profile, lung function, stress and quality of life in sedentary adults: A study protocol for a randomized controlled trial

**DOI:** 10.1371/journal.pone.0263455

**Published:** 2022-02-03

**Authors:** José Pedro Ferreira, Pedro Duarte-Mendes, Ana M. Teixeira, Fernanda M. Silva

**Affiliations:** 1 Research Unit for Sport and Physical Activity (CIDAF, UID/PTD/04213/2019), Faculty of Sport Sciences and Physical Education, University of Coimbra, Coimbra, Portugal; 2 Department of Sports and Well-Being, Polytechnic Institute of Castelo Branco, Castelo Branco, Portugal; 3 Sport, Health & Exercise Research Unit (SHERU), Polytechnic Institute of Castelo Branco, Castelo Branco, Portugal; Prince Sattam Bin Abdulaziz University, College of Applied Medical Sciences, SAUDI ARABIA

## Abstract

**Background:**

Both physical inactivity and sedentary behavior are considered modifiable risk factors for chronic diseases and all-cause mortality. Adult office-workers spend most of their working day in sedentary behaviors, so they are particularly at high risk of developed chronic diseases (e.g., cardiovascular diseases, metabolic disorders like diabetes mellitus, …). It seems important to promote behavioral changes that could prevent or delay metabolic disease development. Evidence supports the use of exercise programs, however, to date there are several knowledge gaps and inconsistencies in the literature regarding the effects of Combined Training (i.e., aerobic plus resistance training) in sedentary healthy adults. This paper outlines an RCT designed to evaluate the effects of a 16-week combined training program on biochemical and immune markers of metabolic disease, lung function, salivary stress hormones and subjective quality of life (primary outcomes), as well as on body composition and physical fitness (secondary outcomes) in sedentary middle-aged office-workers. Furthermore, we aimed to assess the associations between the changes promoted by the exercise program and the different variables studied.

**Methods and design:**

This is a single-blinded two-arm RCT with parallel groups. A minimum of healthy 40 office-workers aged 40–64 years will be recruited to engage in a 16-week intervention study. After baseline assessments, participants will be randomized to one of the two groups: (1) combined training group or (2) control group. Baseline assessments will be repeated after 8 weeks of intervention (mid-testing) and upon completion of the intervention (post-testing).

**Discussion:**

This RCT involves a multi-disciplinary approach and seems to be a relevant contribution to understanding the potential role of combined training in improving the metabolic profile, lung function, stress, and quality of life in adults. The results can provide important insights for clinical recommendations and for the optimization of strategies to prevent metabolic disorders in adults with sedentary jobs.

**Trial registration:**

This trial is registered with ClinicalTrials.gov (registration number: NCT04868240; date of registration April 30, 2021).

## Introduction

The increased urbanization and technological advancements have been accompanied by a high prevalence of sedentary behaviour (SB) and physical inactivity (PI) worldwide, especially in the workplaces [1, 2]. A recent review found that working adults spent about 60% of their waking time in SB and about 4% of the day in moderate to vigorous physical activity (PA) [3]. When compared to other occupations, office-workers had more time spent on SB and less time spent in light PA, both at work as during the wakeful hours [3]. These behavioural patterns have important implications for health since they are associated with many chronic non-communicable diseases (NCDs) [4–6] and even premature death in adults [5–9]. More specifically, the lack of regular PA and the high prevalence of SB promote visceral fat accumulation and hence an increased chronic low grade inflammation and the onset of metabolic disorders, including metabolic syndrome [10–12] that represents a clustering of risk markers (like abdominal obesity, hyperglycaemia, hypertriglyceridemia, low high-density lipoprotein cholesterol (HDL-C), insulin resistance (IR) and pro-inflammatory status) [13], strong and independent contributors to the development of type 2 diabetes mellitus (T2DM) and cardiovascular diseases [14, 15]. A recent study of 2.497 middle-aged adults [16] showed that for each additional hour daily of SB during waking time, the odds of developing T2DM and metabolic syndrome increased by 22% and 39%, respectively. According to Leong et al. [17], T2DM is preceded by IR years before symptoms arise, and not all individuals with obesity and genetic predisposition develop this disease. Thus, it seems important to identify those at risk for IR and other risk factors (e.g., abdominal obesity, chronic systemic inflammation) at an early stage [18]. In addition to the negative impact on metabolic health, an inactive lifestyle also accelerates the age-associated decline in lung function [19–21]. In turn, reduced lung function has been associated with an increase of chronic non-communicable diseases (NCDs) and death [19, 22]. The mechanisms through which high levels of SB may affect lung function are not well understood, however, some studies speculate that inflammation may be a biological link [20]. Stress and health-related quality of life (HRQoL) are also important components of health [23] and studies showed that, in the work context, the time in occupational sitting can be associated with increased stress (i.e., salivary cortisol (s-COR) levels) [24–26].

Considering the levels of sedentariness and physical inactivity in office workers, and its negative impact on health, it seems vital to promote behavioural changes that could prevent or delay metabolic disease development, as well as improve lung function, stress, and HRQoL. Regular exercise has been proposed for the prevention and treatment of many chronic NCDs, including T2DM since it promotes metabolic and immune changes [11, 27, 28]. Its protective health effects can be attributed to the anti-inflammatory effects of exercise, mediated by protection against visceral fat accumulation, increasing the secretion of anti-inflammatory cytokines, such as interleukin (IL)-10, IL-1ra and serum adiponectin levels, and decreasing the pro-inflammatory cytokines like tumor necrosis factor-alpha (TNF-α), IL-1β, IL-6 and serum leptin levels [11, 27, 29]. Although moderate evidence supports the use of exercise programs to prevent or reverse metabolic disorders, to date, there are several knowledge gaps and inconsistencies in the literature regarding the optimal dose and type of exercise [30–32]. Aerobic exercise (AE) has been deemed effective to improve metabolic profile (e.g., reduce IR and increase anti-inflammatory effects) [33–35]. Evidence regarding resistance exercise (RE) is not as strong, but besides increasing muscle strength, it can also induce beneficial changes on some metabolic risk factors [36–38]. However, whether a combined training (CT), combining AE and RE, may be more effective and provide an additive effect in promoting a better metabolic profile in sedentary adults remains to be determined. Some studies have demonstrated that CT improves body composition [32, 39–41], inflammatory cytokines [32, 41], IR [39, 41, 42] and other metabolic disease risk factors [32, 39, 40, 43]. However, these studies have had one or more of the following methodological confounds: vast breadth of populations (i.e., elderly) [40, 42], co-morbidities included (i.e., T2DM) [32, 39, 43] which may limit generalizability to the adults that are healthy; and short intervention durations or lack of a control group [39, 41]. Thereby, and according to a recent meta-analysis [31], more studies with CT are required to improve the quality of evidence on this topic. Regarding lung function, cross-sectional studies have shown a positive association between PA levels and lung function among middle-aged adults without a respiratory disease [20, 21], however, causal inferences cannot be made. To our knowledge, the effects of a long-term CT exercise program on the lung function of healthy adults have not been examined, with the published studies focusing on adults with obstructive lung diseases [44]. The effectiveness of a long-term CT program in aspects of stress and HRQoL are also not well established, however, some studies suggest that PA is beneficial on the regulation of stress [24, 25]. The s-COR and alpha-amylase (s-AA) are the proxy markers of the two main stress response systems [45] and could be used to assess the stress levels of worker adults, increasing knowledge on the effects of a CT program on these indicators.

Unfortunately, the high methodological confounds of some of the previous studies and knowledge gaps lead to little evidence regarding the effectiveness of CT to improve the biochemical and immune markers of metabolic disease, lung function, quality of life, and stress in sedentary adult office workers. So, new studies are required to improve the quality of evidence on this topic. This trial aims to analyse the effects of a 16-week CT program on metabolic disease risk markers, including inflammatory cytokine profile, markers of IR (fasting glucose and insulin levels, HOMA-IR), leptin and adiponectin levels (as surrogate markers of adipose tissue dysfunction), lung function, salivary stress hormones and subjective quality of life (primary outcomes), as well as body composition and physical fitness (secondary outcomes), in sedentary middle-aged office workers of both genders. Furthermore, we aimed to assess the associations between the changes promoted by the exercise intervention and the different variables studied.

## Materials and methods

### Study design and setting

**Fig 1:**
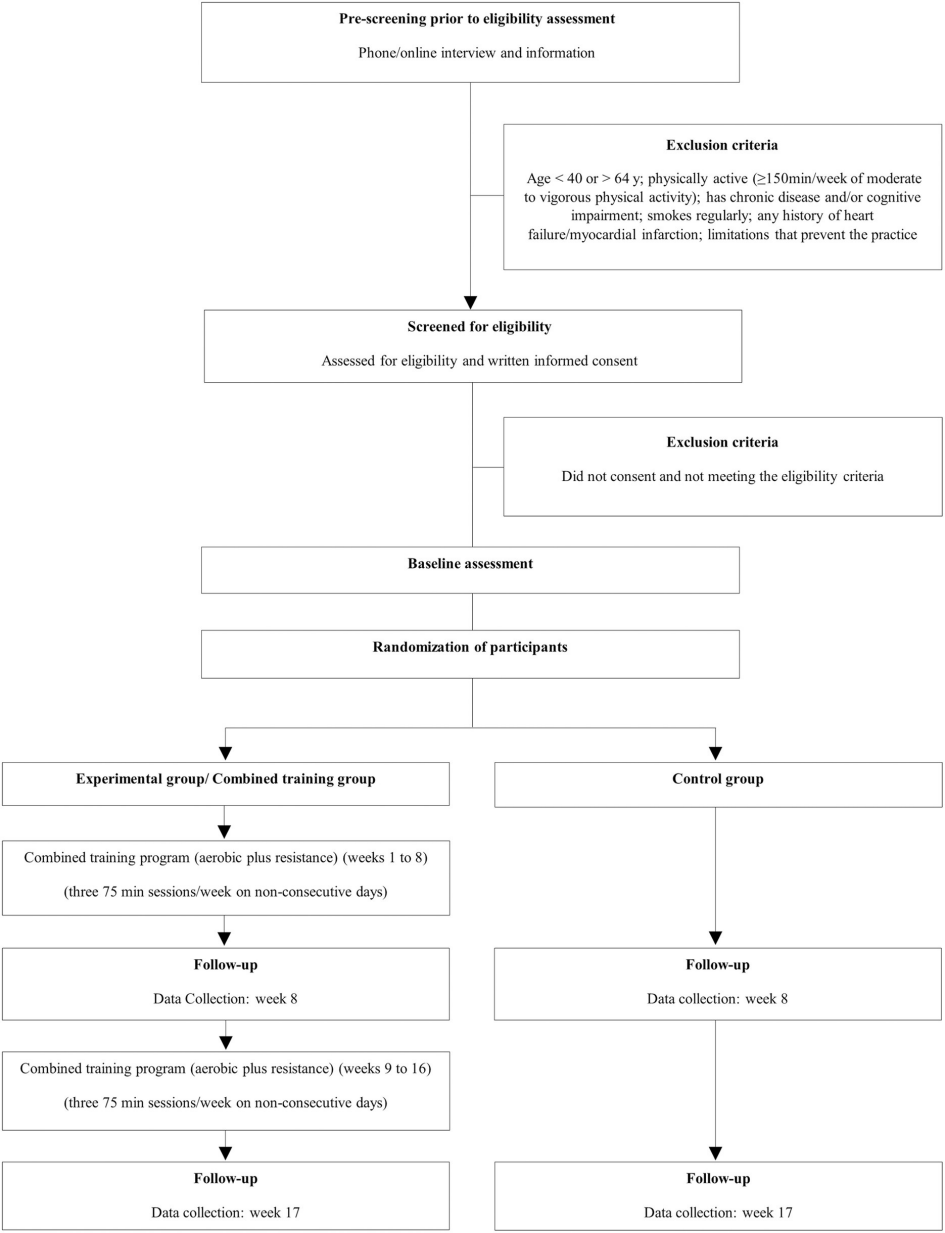
Study design diagram.

This trial is designed as a single-blinded two-arm randomized controlled trial (RCT) with parallel groups, and an allocation ratio of 1:1. Fig 1 illustrates the overall study. This study protocol follows the SPIRIT Statement guidelines for standard protocol items in clinical trials [46] and is registered in the Clinicaltrials.gov ID: NCT04868240 (date of registration April 30, 2021). This RCT study has been designed and results will be reported according to the Consolidated Standards of Reporting Trials (CONSORT) statement [47]. Furthermore, and since that exercise training interventions are not properly and completely addressed by the CONSORT checklist [48], the guidelines of the Consensus on Exercise Reporting Template (CERT) will also be considered [49].

### Participants

In this RCT, a minimum of 40 office-workers aged 40–64 years will be recruited, enrolled, and randomized (n = 20 per group). Potential eligible subjects will be recruited from two office companies in Coimbra city, Portugal. The principal investigator (PI) of this project will meet with the directors/leaders of these companies to present and discuss the purposes and procedures of this research project. Afterwards, a poster will be disseminated on their internal web pages and the subjects will be able sign up online or by telephone. Interested subjects will receive information about the study and will be pre-screening for initial eligibility criteria through telephone/online interviews. The subjects ruled eligible will be invited for an in-person screening visit where they learned more about the study, sign the informed consent form, and complete additional screening measures. Lastly, the subjects that meet all the defined eligibility criteria will be invited to return for a baseline assessment visit. After the baseline assessments, participants will be randomized and notified by email/telephone (by the PI).

Participant inclusion and exclusion criteria are presented in Table 1. These criteria are conceived to recruit healthy but physically inactive adults, with occupational jobs characterized by low levels of PA and high levels of SB and exclude adults with poor health or physically active. The term “physically inactive” refers to adults who do not achieve the recommended levels of moderate to vigorous PA outlined in the “WHO—Global recommendations on physical activity for health” [50]. These recommendations include the weekly accumulation of at least 150 minutes of moderate-intensity aerobic activity or 75 minutes of vigorous-intensity aerobic activity, and muscle-strengthening activities at moderate or vigorous-intensity on two or more days per week [50, 51]. The protocol of this study has been approved by the Ethical Committee for Health of the Faculty of Sport Sciences and Physical Education of the University of Coimbra (reference: CE/FCDEF-UC/00512019; approved on June 09, 2021). All procedures will be conducted in accordance with the Declaration of Helsinki [52] as well as with data protection and security regulations [53]. Study procedures, potential risks and expected results will be explained to the participants that will also hear of the voluntary character of their participation in this study, and of their right to withdraw without any prejudice or consequence.

**Table 1:** Inclusion and exclusion criteria for participants.

**Inclusion Criteria**
Men and women between 40 to 64 years old
Physically inactive adults (not meeting the Global recommendations on physical activity for health [50, 51])
Full-time workers with sedentary occupations (report spend ≥65% of their workday in activities with low energy expenditure i.e., 1.5 METs or lower);
Body mass index between 18 and 29.9 kg/m^2^
Willingness to maintain the current nutrition pattern and to participate in all testing assessments
**Exclusion Criteria**
Diagnosis of chronic disease (e.g., diabetes mellitus, cardiovascular and pulmonary diseases)
Cognitive or psychiatric conditions that need chronic treatment (e.g., depressive disorder) and that in the investigators’ opinion could interfere with the study outcomes
Take any regular medication that in the investigators’ opinion could interfere with the study outcomes
Limitations that prevent them from practicing exercise
History of heart failure and/or myocardial infarction
Uncontrolled or abnormal blood pressure
Participation in any intervention trial within 6 months prior to screening
Current participation in another intervention trial
Smoking
Failure to provide consent

### Randomization

Participants who meet all eligibility criteria will be randomly assigned to either control or experimental group with a 1:1 allocation as per a computer random number generator. Permuted block randomization will be used to ensure an equal sample size in the two groups. The block sizes will not be divulged to ensure concealment. This randomization process will be generated by an independent biostatistician. The study participants will be enrolled and assigned to their respective groups by the PI. The experimental group will perform 16-weeks of CT exercise and the control group will maintain their usual habits, including not participating in any exercise program.

### Estimated sample size

The sample size was calculated using a priori power analysis through the G*Power software version (3.1.9.2.), following the guidelines of Faul et al. [54]. For an effect size of 0.30, a sample size of 20 participants per group achieves 95% power to detect differences among the means versus the alternative of equal means using an F test with a 5% α-level. This analysis indicated a minimum total sample size required in this study. We plan to enrol 20% more persons to account for dropouts.

### Exercise intervention

The exercise program will be conducted following the American College of Sports Medicine (ACSM) recommendations [29, 55]. The exercise sessions will occur 3 times/week (i.e., Monday, Wednesday, and Friday) during approximately 75 min/session over 16 weeks (achieving 225 min/week, meeting public health guidelines for middle-aged adults), in an enclosed gymnastic pavilion. All exercise sessions will be conducted and supervised by two instructors who graduated in Sport Sciences with wide experience in the area. The two instructors are also responsible for encouraging participants to complete the exercise sessions/program, provide feedback, and assure the correction and safety of exercise execution. Before starting the exercise program, subjects will be submitted to estimated maximal dynamic strength tests aiming at setting their workload (i.e., 10 repetitions maximum (RM) protocol) [56].

Each session will consist of a warm-up (10 min), the main part of the session (aerobic plus resistance exercise) (60 min), and a return to calm (5 min). In the warm-up, the participants will perform 10 minutes of light-to-moderate intensity cardiorespiratory and muscular endurance activities to increase muscle temperature and promote an adequate cardiovascular response for CT exercise [55]. The main part of the session will be divided into two phases. In phase 1, participants will perform eight resistance exercises, that involve the use of body weight and free weights, namely: chest press, lateral arm raises, bent-over two-arm row, push-ups (incline or flat), front squat, deadlift, calf raises, and abdominal exercises (e.g., planks with knee and elbows or knees and toes). The participants will perform 1–2 sets of 6–15 repetitions with a 2–3 min rest interval between sets and exercises. For free weights exercises, the load will be adjusted individually to work between 45–90% of the estimated 1-repetition maximum (1RM), following a gradual progression in intensity throughout the weeks (see Table 2) [55]. The constant load method and the progressive load method will be used in the program. Participants will be instructed to inhale and exhale during the eccentric phase and concentric phase, respectively, while maintaining a fixed cadence of movement at a ratio of 2:2 s [55]. Maximal dynamic strength will be re-evaluated after 8 weeks (mid-testing) using 10RM protocol to guarantee those exercise prescriptions are continuously updated. In phase 2, participants will perform aerobic exercises (e.g., fast walking, running) with intensity based on predicting heart rate reserve (HRR). Aerobic exercise prescriptions will be based on 60%-95% of HRR following a gradual progression in intensity throughout the weeks that can be continuous, interval, or varied continuous (Table 2). Resting heart rate (HR) will be reassessed every 4 weeks so that HRR prescriptions can be continuously updated throughout the CT program. The participants will also be asked to rate their perceived effort levels on the Borg CR-10 scale to ensure that they are exercising at the planned intensity. In the last 5 minutes of the session, a cool-down period involving aerobic activities of light intensity, relaxation, and static stretching movements of the large muscle groups (10–15 seconds in each position and 1–2 repetitions) will be performed to allow for a gradual recovery of HR and blood pressure (BP) [55].

**Table 2:** Overview of the exercise program.

Group	Frequency and duration	Intensity				Types
		(Week 1–4)	(Week 5–8)	(Week 9–12)	(Week 13–16)	
Combined Training Group	3 times per week (Monday, Wednesday, and Friday) Main part of the session: 60 min (30 min aerobic plus 30 min resistance)	Resistance exercises: 1–2 sets of 10–15 reps at 45%-65% 1RM; and RPE of 5–6 on the Borg CR-10 scale (from 0 = extremely easy to 10 = extremely hard).	Resistance exercises: 2 sets of 10–12 reps at 60%-75% 1RM; and RPE of 5–6 on the Borg CR-10 scale.	Resistance exercises: 2 sets of 8–12 reps at 70%-85% 1RM; and RPE of 6–7 on the Borg CR-10 scale.	Resistance exercises: 2 sets of 6–12 reps at 75%-90% 1RM; and RPE of 7–8 on the Borg CR-10 scale.	Loading methods: constant and progressive. Examples: Chest press, lateral arm raises, bent-over two-arm row, push-ups, front squat, deadlift, calf raises, abdominal exercises.
		Aerobic exercises: 60%-75% of maximum HRR; and RPE of 5–6 on the Borg CR-10 scale.	Aerobic exercises: 70%-85% of maximum HRR; and RPE of 5–6 on the Borg CR-10 scale.	Aerobic exercises: 80%-90% of maximum HRR; and RPE of 6–7 on the Borg CR-10 scale.	Aerobic exercises: 80%-95% of maximum HRR; and RPE of 7–8 on the Borg CR-10 scale.	Exercise continuous, interval or varied continuous. Examples: fast walking, running, stepping, circuit training.
Control Group		No exercise training				

Notes: min, minutes; HRR, heart rate reserve; reps, repetitions; RPE, rate of perceived exertion; 1 RM, 1-repetition maximum.

Participants from both groups will be encouraged to conduct their daily activities as usual outside of the study and to maintain the same nutritional pattern/diet. The participants may not participate in any other exercise program. For ethical reasons and to avoid possible dropouts [48] during the study period, the control group will also receive the exercise intervention after having served as a control group.

#### Program attendance/adherence

The exercise program will last for a total of 16 weeks, with a total of 48 exercise sessions. The percentage of adherence to the exercise sessions will be calculated individually through the total sum of sessions participation. Participants of the CT group will be invited to participate in all training sessions and only those who have an adherence of ≥ 80% will be integrated into the analysis (i.e., attends 38 of the 48 exercise sessions) [57, 58]. Attendance at sessions will be recorded and entered on a database. Strategies to promote exercise adherence will include texts and email support as suggested by Connelly et al. [59]. If the participant has two consecutive absences, he or she will be contacted to return to the physical exercise sessions, since the lack of adherence may impair obtaining the desired intervention effects [60].

#### Exercise intensity control

Heart rate monitors (Polar V800^®^, Polar Electro Oy, Finland) will be used during all sessions to assess, monitor, and adjust the intensity of the HR interval of the participants. As a preventive measure, the exercise intensity will be indirectly predicted applying Karvonen’s formula to predict target HR [61]:

TargetHR=((MaximumHR–RestingHR)%intensity)+RestingHR))

Maximum HR (HR_max_) will be calculated using the equation formula proposed by Gellish et al. [62]:

HRmax=((207–(0.7xage))

For resistance exercises with free weights, the load will be adjusted individually through an estimated 1RM. Thus, the 10RM protocol will be performed for all exercises with free weights [56] and will be re-evaluated after 8 weeks to guarantee that the exercise prescription is continuously updated. As a complementary method, the intensity will also be monitored through the rate of perceived exertion (RPE) using a reliable and valid tool [63] − Borg CR-10 scale [64]. This scale will be presented at the end of each session, in which the participants record their RPE (range from level 1 to 10, extremely easy and extremely hard, respectively) [64].

### Adverse events

Despite all the safety procedures established to ensure the safety of the participants (e.g., exclude those with significant risk in participating; control and measurement of training intensity), unforeseen adverse events may occur. An adverse event represents an untoward medical problem during the study, which may or may not be caused by the exercise program or the assessments [46]. Exercise instructors will monitor adverse events, asking the participants if they have experienced any health problems (e.g., physical injuries, muscle soreness) since the last session [58]. All adverse events that may occur during the participation in this study will be monitored and analysed by the research team.

### Blinding (masking)

Given the nature of this program, neither participants nor PI can be blinded to allocation but are clearly instructed to not reveal the allocation status at the follow-up assessments [46]. To minimize the risk of bias, we will adopt the following methods. The randomization process will be generated by an independent biostatistician using a computer-generated list of random numbers. Participants will be enrolled and assigned to their intervention groups by the PI. The assessments will be organized by the PI and will be applied by invited specialists and co-investigators of the research team, which will be blinded to allocation. The two instructors of the CT exercise program will not take part of the data collection. To minimize differences in the procedures, the same testing staff will perform data collection in the different assessment moments (pre-, mid- and post-testing). Data will be entered and organized electronically into the database by an independent researcher outside the research team. All participant information will be coded, and the code will be stored in a locked local and only the PI will have key access. This procedure assures that both outcomes assessor and data analyst will not have access to information about the allocation of participants [46].

### Outcome measures

This trial includes a screening and baseline assessment period of 20 days (four visits), a 16-week CT program period, and a mid-testing and post-testing period of 3 (one visit) and 10 days (two visits), respectively. On the first visit, subjects will sign the informed consent form and will complete additional screening measures. Subjects that meet all eligibility criteria will be enrolled and complete a set of outcome measures over visits 2 and 3. After the baseline assessments, subjects will be randomly assigned to either control or experimental group, and the participants of the experimental group will carry out the evaluations of 10RM (Visit 4). The exercise sessions will be performed over 16-weeks on a single site and supervised by two instructors. Baseline assessments will be repeated after 8 weeks (mid-testing, visit 5) and upon completion of the intervention (visits 6 and 7). Importantly, we will consider the phase of the menstrual cycle in non-menopausal women (i.e., menstrual phase, follicular phase, ovulation phase and luteal phase), taking baseline and follow-up evaluations at the same time of the cycle. A timetable of the enrolment, intervention and assessments are presented in Table 3.

**Table 3:** Schedule of enrolment, interventions, and assessments.

	STUDY PERIOD							
	Baseline				Intervention	Follow-ups		
Timepoint	Visit 1^a^	Visit 2^a^	Visit 3^a^	Visit 4^a^	Sessions 1–48^b^	Visit 5^c^	Visit 6^d^	Visit 7^d^
Day number	-20 to -15	-15 to -10	-10 to -5	-5 to -1	1 to 112	56 to 58	113 to 118	118 to 123
**SCREENING/BASELINE**								
Informed consent, review inclusion/exclusion criteria	x							
Sociodemographic information	x							
Medical history	x							
Body mass index measurement	x							
**INTERVENTIONS**								
Combined training group					x			
Control group					x			
**ASSESSMENTS**								
Fasting blood samples		x					x	
Saliva samples		x					x	
Body composition			x			x		x
Physical fitness			x			x		x
Lung function (Spirometry)			x			x		x
Subjective quality of life			x			x		x
Systolic and diastolic blood pressure	x		x			x		x
Assessment of dietary intake			x					x
Accelerometry		x					x	
**RANDOMIZATION**				x				
Maximal dynamic strength to estimated 1RM				x		x		

^a^ Screen and baseline assessments will be completed within 20 days;

^b^ The exercise sessions will be performed 3 times/week (i.e., Monday, Wednesday, and Friday) over 16 weeks;

^c^ This study includes an intermediate assessment, which will be carried out in the eighth week of intervention;

^d^ Post-testing assessments will be done within 10 days of the last exercise session.

#### Primary outcomes

*Biochemical assessment I*: *Fasting blood samples*. Blood collection will be performed in a seated position from the antecubital vein, at rest after a 12-hours overnight fast, and the blood samples will be aliquoted into kEDTA and serum tubes. The participants will be instructed to avoid extreme physical efforts 24 hours before the blood collection. Determination of blood counts will be done immediately after the blood collection, using an automated Coulter Ac•T diff Hematology Analyzer (Beckman Coulter^®^, Inc, USA). Biological samples will be centrifuged at 1500xg for 10-min and plasma and serum samples will be frozen at -80°Celsus (C) for posterior analysis. The levels of insulin, glucose, glycated haemoglobin (HbA1c), total cholesterol, low-density lipoprotein cholesterol (LDL-C), HDL-C, triglycerides, cytokines IL-1β, IL-1ra, IL-6, IL-10, transforming growth factor beta 1 (TGF-β), TNF-α, adiponectin and leptin will be analysed. Fasting glucose, triglycerides and cholesterol levels will be determined by standard enzymatic assays (ABX Pentra, Germany). Insulin, leptin and adiponectin will be analysed by ELISA (R&D Systems, UK). The Homeostatic model assessment (HOMA-IR) will be calculated using the Matthews formula [65]:

$$HOMA-IR=\frac{fasting\;glucose\;[mmol/L]\;x\;fasting\;insulin\;[\mu U/l]}{22.5}$$

Inflammatory cytokines will be analysed by ELISA kits (Thermofisher, UK) according to the manufacturer instructions. All blood collections for the biomarker analysis will be performed in the morning (between 07:30h – 10:30h) by a registered nurse.

*Biochemical assessment II*: *Saliva samples*. Saliva will be collected through a gold standardized method for biological assays—passive drool—which requires participants to “expel” saliva (let the saliva drop) into polypropylene tubes [66]. The participants will be comfortably seated with their head slightly lowered, allowing spontaneous saliva flow to accumulate in their mouth [67]. Before saliva collection, the participants will be instructed to rinse their mouth for 60 seconds with water to remove any food residues and then wait at least 10 minutes [67, 68] before saliva collection. Moreover, according to procedures in other studies, the participants will be required to avoid vigorous physical exercise 24h before the collection, alcohol for 12 hours, brush teeth or eat a major meal for 60 minutes, and foods with high sugar, acidity, or caffeine immediately before sample collection [67, 68]. After collection, saliva samples will be stored frozen at −20°C until assayed. The saliva samples will be subject to a single freeze-thaw cycle. On the day of the assay, samples will be centrifuged to remove particulate matter, and the salivary levels of cortisol (s-COR), α-amylase (s-AA), immunoglobulin A (s-IgA), and Lysozyme (s-Lys) will be analyzed by commercial kits. S-COR, s-IgA, and s-Lys will be analyzed by ELISA (Salimetrics, USA) and s-AA by a kinetic reaction assay (Salimetrics, USA), according to the manufacturer instructions. All saliva collections for biomarker analysis will always occurred at the same time in the morning to minimize circadian effects (between 09:30h – 10h30).

*Brachial blood pressure*. The right and left brachial systolic blood pressure (SBP) and diastolic blood pressure (DBP) will be measure using an automated oscillometric cuff (OMRON, HEM 907, Japan). The participant will be comfortably seated, with the legs uncrossed and the back and arm supported, for 3–5 minutes without speaking and/or moving before recording the first measure [69, 70]. Two readings will be taken at intervals of at least 1–2 minutes and the average of those readings will be recorded as the participant’s BP. However, if the values deviate by ≥ 5 mmHg, a third measurement will be performed, and the average of these multiple readings will be used [69, 70]. The participants must avoid caffeine, physical exercise, and smoking for at least 30 minutes prior to measurement [69]. The brachial BP will be assessed in the morning (between 09:00h – 12:00h) and will be performed by a doctor.

*Lung function*. Spirometry will be conducted using the Spiropalm 6MWT (Cosmed, Italy) Spirometer. Participants will perform a maximal inspiratory and expiratory maneuver to achieve the Forced Vital Capacity (FVC), Forced Expiratory Volume in 1 second (FEV_1_), and Peak Expiratory Flow (PEF). Participants’ data will be classified to indicate repeatability. In this sense, the participants will have to complete at least three acceptable FVC maneuvers to be included in the analysis (i.e., the difference between the best two FEV_1_ and FVC values must be within 150 mL) [71, 72]. The best FEV_1_ and best FVC measures of the participants will be recorded and used for data analysis, according to procedures adopted in other studies [20, 21]. Predicted FEV_1_ (FEV_1_% pred), predicted FVC (FVC% pred), and predicted PFE (PFE% pred) will be calculated based on age, stature, ethnicity, and sex using the formulas developed by the Global Lung Function Initiative [73]. The lung function assessment will occur in the morning (between 09:00h – 12:00h) and will be performed by a doctor.

#### Subjective health-related quality of life (HRQOL)

*World health organization quality of life instruments–BREF*. The World Health Organization Quality of Life Instruments–BREF (WHOQOL-BREF [74]) questionnaire, validated for the Portuguese population [75], assesses the subjective quality of life. WHOQOL-BREF includes 26 questions (with structured responses on a 5-point Likert scale) of which two are more general, related to the general perception of the quality of life and the general perception of health, and the remaining 24 comprise physical, social relations, environment, and psychological domains [75, 76]. To calculate the final score, it is necessary to invert items 3, 4 and 26. The final score should be converted into a scale from 0 to 100, with a higher score corresponding to a better perception of quality of life [75, 76].

*Satisfaction with life scale*. The Satisfaction with Life Scale (SWLS [77]), validated for the Portuguese adult population [78], assesses a person’s global judgment of life satisfaction. SWLS comprises 5-item formulated in the positive sense, with structured responses on a 7-point Likert scale (range from level 1 to 7, strongly disagree to strongly agree, respectively) [77, 78]. Final results range between 5 (low satisfaction) to 35 (high satisfaction) [77].

*Perceived stress scale*. The Perceived Stress Scale (PSS [79]), validated for the Portuguese adult population [80], measures the person’s life situations, assessed as stressful. The 14-items on the scale are created to assess the degree to which subjects believe that their life is unpredictable, out of control and overloaded, during the previous month [80]. PSS also includes several direct queries about current levels of experienced stress [80]. Of the 14-items, seven are considered negative and the other seven as positive, all rated on a 5-point Likert scale [80]. To calculate the final score, it is necessary to take into attention that items 4–7, 9, 10 and 13 refer to positive situations, being necessary to invert the quotation [79, 80]. Results range between 0 to 56 points, with a higher score representing higher levels of stress [80].

*36-item short form survey*. The 36-Item Short Form Survey (SF-36 [81]), validated for the Portuguese adult population [82, 83], is a generic instrument to evaluate HRQoL. This instrument comprises 36 questions which cover 8 subdomains of health, namely: physical functioning (PF), role-physical (RP), role-emotional (RE), bodily pain (BP), general health (GH), vitality (VT), social functioning (SF), and mental health (MH) [81–83]. These 8 subdomains can be grouped into two component scores, namely: a physical component summary and a mental component summary. Dimension scores are presented on a positively oriented scale from 0 (poor health status) to 100 (good health status). Higher scores on both subscales represent better health and functioning [81].

#### Secondary outcomes

*Anthropometry and body composition*. Body mass will be assessed using a portable scale (SECA 761, Germany) with accuracy to the nearest 0.1 kg while wearing minimal clothes. Stature will be determined using a portable stadiometer (Seca Bodymeter 208, Germany), with accuracy to the nearest millimeter, according to the standardized procedures [84]. The body mass index (BMI) will be calculated according to the usual formula: BMI = (weight/height ^2^). Waist circumference (WC) will be assessed twice using a flexible tape measure (Hoechstmass-Rollfix, Germany) made of fiberglass, with an accuracy of 0.1 cm and according to the standardized procedures of the National Institute of Health [85]. If the two WC measurements differ by ≥ 0.5 cm, a third measurement is taken, and the mean circumference will be calculated from the two nearest measures. Values of skeletal muscle mass (kg), fat mass (kg), bone mineral mass (%), and body fat (%) will be determined using the tetrapolar bioimpedance (Inbody 270, USA). The participants age, sex, and stature will be entered into the bioimpedance scale. The impedance measurements will be performed according to the literature [86], removing the watch or any other metallic object from the participants. These measures will be conducted between 09:00 and 11:00 a.m. by invited specialists and co-investigators of the research team.

*Physical fitness*. ***Cardiorespiratory fitness (CRF)***. The “Chester Step Test” will be conducted using audio (with stepping beat rhythms) and a step (height, 15, 20, 25, or 30 cm, depending on the age and physical ability of the subject [87, 88]). The participant is required to step to the beat rhythm at 15 steps/min for 2 minutes following which HR and RPE are recorded (Level 1) [87]. Every 2 minutes the stepping rate increase slightly by 5 steps/min, and the HR and RPE are again recorded [87]. The test continues in this progressive mode until the participant achieves 80% of their HR_max_ or reports a moderately vigorous level of exertion (RPE = 14) [88]. The maximum test duration is 10 min, which corresponds to the last level of the test (i.e., Level 5). Maximal oxygen consumption (VO_2_max) will be calculated using the CST software (Cartwright fitness, UK) [88].

***Muscle strength***. “Hand Grip Test”–Handgrip strength will be assessed using a hand dynamometer (Jamar, Lafayette Instrument Company, USA) to the nearest 0.1 kg. The participant starts by adjusting the grip bar, so the second joint of the fingers fits under the handle [55]. Measurements will be taken with the participant standing with arms parallel to the body. The participant squeezes the handgrip dynamometer as hard as possible, without holding the breath [55]. The handgrip test will be repeated twice in each hand and the final score will be the highest of the two readings (to the nearest kilogram) for each hand added together [55].

***Arms flexibility***. “Back Scratch test”–This test was developed by Rikli and Jones [89] to measure flexibility in the shoulder joint and shoulder arch on the right and on the left side. The participant will place a hand over the shoulder of that same arm in the direction of the floor, and the other hand up the middle of the back in the direction of the head [89]. Then, the participant tries to touch or overlap the fingers of both hands. The distance in centimeters (+ or -) between the extended middle fingertips of each hand will be registered [89]. Two attempts will be taken on each side (right and left arm over), and the final score will be the mean of the two measures (to the nearest half cm) [89].

***Legs flexibility***. “Modified sit-and-reach test”–This test was developed by Hoeger and Hopkins [90] to evaluate hamstring and low-back flexibility. This test requires a sit-and-reach box (height, 30.5 cm) and a ruler (range of 0 to 70 cm). The participant sits on the floor with the head, back and hips in contact with the wall and the feet with the sit-and-reach box [90, 91]. Participants will be asked to overlap hands (with middle fingers on the same level) and to execute a scapular abduction to reach out level with the measurement scale. Then, the ruler is moved along the box until the zero point of the ruler is even with the tip of the fingers—finger to box distance (FBD) [90, 91]. FBD represents a relative zero point for each subject based on proportional differences in limb lengths [90]. With FBD established, the participant slowly leans as far forward as possible, allowing the head and shoulders to stop having contact with the wall and the fingers to slide over the ruler [90]. The distance between the “0 cm” point and the end point will be recorded [90]. Two measurements will be performed, and the final score will be the mean of the two measures (nearest half cm). All measures of physical fitness will be conducted between 09:00 and 11:00 a.m. by invited specialists and co-investigators of the research team.

#### Supportive measures

*Levels and patterns of physical activity and sedentary behaviour*. Sedentary time and PA levels (light-, moderate-, and vigorous intensity) will be assessed using a triaxial accelerometer (Actigraph GT3X, Actigraph Corporation, Florida, USA). Accelerometers will be initialized to capture data at 100 Hertz using ActiLife software V6 13.3 (ActiGraph, Florida, US), and the data will be recorded in 60-s epochs [92]. Participants will be instructed to wear the device on their waist near the right iliac crest during all waking hours for 7 consecutive days (i.e., 5 weekdays and 2 weekend days), except when sleeping, bathing, or swimming [92, 93]. Non-wear time will be defined as ≥ 60 minutes of consecutive zero accelerometer counts (allowing up to 2 minutes with limited movement) [92, 94]. All participants with at least 4 days with 600 min (10 h) of wear time per day will be included in the analysis [92, 94, 95]. To classify accelerometer output data into different SB and PA intensity categories, the following cut-offs will be used: sedentary behavior (accelerometer vector magnitude < 200 cpm), LPA light physical activity (200–2689 cpm) and moderate-to-vigorous physical activity (≥ 2690 cpm) [96].

*Maximal dynamic strength (estimated 1 RM)*. Participants of the CT group will perform incremental loading tests to estimate 1RM in all exercises with free weights, i.e., chest press, lateral arm raises, bent-over two-arm row, front squat, deadlift, and calf raises, preceded by a standardized general warm-up [56]. Participants will warm up by performing 2 x 10 reps of each exercise, using light loads to avoid injuries. We will use the protocol for 10RM testing following the National Strength and Conditioning Association guidelines [56]. After the completion of the warm-up sets, the instructors will adjust the loads so that the 10RM can be measured within 3 to 5 testing sets [56]. The process of testing will continue until a load allowing only 10 RM is determined [56]. This protocol will be conducted between 09:00 and 12:00 a.m. by two experienced instructors. The calculation of 1RM will be achieved based on the mathematical equation proposed by Brzicky [97]:

$$1RM=\frac{\left(100\;x\;rep\;wt\right)}{\left(102.78-2.78\;x\;number\;of\;repetitions\right)}$$

*Assessment of dietary intake*. Dietary habits will be assessed using a semi-quantitative Food Frequency Questionnaire (FFQ) [98], based on the previous 12 months. The FFQ was previously adapted for the Portuguese population [99, 100]. This instrument contains 82 food items, including 9 possible frequency responses, ranging from “never” to “six or more times per day” [100]. Any foods not reported in the FFQ but eaten regularly can be registered in an open-ended section [100]. To obtain nutrient intake, the frequency of consumption of each food item will be multiplied by the respective standard average portion (in grams), and by a seasonal variation factor for food consumed in specific seasons (0.25 are considered the average seasonality of three months) [100]. The conversion of food into nutrients will be performed using the software program Food Processor Plus (version 5.0, ESHA Research, Salem, OR), based on values from the US Department of Agriculture and from the composition of typical Portuguese foods that were included in this software [100, 101].

*Health*, *demographic*, *and lifestyle data*. Sociodemographic characteristics such as chronological age, gender, education degree, and marital status will be collected to characterize the sample and to adjust models. Health information will include past and current diseases (acute and/or chronic), medication, family history of metabolic diseases, dementia, or cognitive impairment. The “Pittsburg Sleep Quality Index” (PSQI) questionnaire will also be applied, before and after the trial, to assess subjective sleep quality over the previous month [102, 103]. Lifestyle information will include smoking history, consumption of alcoholic drinks, substance abuse and supplement use.

### Data analysis

Data analyses will be performed using SPSS Statistics version 25.0 (SPSS Inc., IBM Company, Chicago, Illinois, USA). Descriptive statistics (mean ± SD) will be performed for all variables in the analysis. The assumption of normality will be tested using histograms, normal probability plots and Shapiro-Wilk test. Chi-squared test will be performed to analyse sex proportions between groups. Assuming data normality, a two-way analysis of variance (ANOVA) for repeated measures will be applied for intra- and inter-group comparisons. In absence of normality, non-parametric Kruskall-Wallis test will be used. Bonferroni’s *post hoc* test will be performed if significant differences exist (alpha level set at 0.05). The magnitude of the global effect size will be calculated using Cohen’s effect size [104]. The Cohen’s d effect size is categorized according to the following criteria: *d* = < 0.20 (small), 0.21–0.79 (medium) and > 0.80 (large) [104]. The bivariate associations between the changes promoted by the exercise program and the different variables studied will be assessed by Spearman’s rank and Pearson’s coefficients. The strength of the associations will be classified as follows [105]: very high (0.90 < r < 1.00); high (0.70 < r < 0.90); moderate (0.50 < r < 0.70); low (0.30 < r < 0.50); little (0.10 < r < 0.30). Significance level set at *p* ≤ 0.05.

## Discussion

This project will allow us to investigate the potential effects of a 16-week CT program on several health indicators, namely: biochemical and immune markers of metabolic disease, body composition, physical fitness, lung function, salivary stress hormones and subjective quality of life. Furthermore, we will assess the associations between the changes promoted by the exercise intervention and the different variables studied. Thus, based on results of other studies with a vast breadth of populations or co-morbidities included [32, 39–41], we will check the hypothetical premise that 16-weeks of CT program, carried out in a moderate to vigorous intensity, can improve the inflammatory status, insulin resistance and other metabolic risk factors in sedentary office workers. We also look at the hypothetical premise that the CT program will improve lung function [20, 21, 44], stress levels and subjective quality of life [24]. Moreover, we will check the premise that some changes promoted by the exercise intervention are associated with different variables studied, for example, evidence suggests that most of the benefits of exercise on the inflammatory cytokines seem to be mediated by body composition changes [11, 32]. Also, a better lung function could be associated with a decrease in pro-inflammatory cytokines (induced by exercise) [106, 107]. This research project involves a multi-disciplinary approach and should be seen as an original and relevant contribution to understanding the potential role of a specific mode of exercise in improving metabolic risk markers, lung function, stress, and quality of life in sedentary adults. The results of this study will provide important insights for clinical recommendations and for the optimization of prevention and treatment strategies to combat metabolic diseases in sedentary adults. In addition to these indicators, de Vries et al. [108] have concluded that exercise may also play an important role in reducing work-related fatigue and improving a set of indicators of employee well-being, such as work ability, cognitive status/ function, and sleep quality. The results of this project may also help to develop further theories and models explaining CT effects in sedentary adults.

### Ethics and dissemination

Formal amendment to the protocol will be required if any alterations to the protocol which impact the conduct trial or the benefits and safety of participants were performed (e.g., changes to study design/aims, participants’ characteristics, study outcomes, instruments, and procedures) [46]. These amendments will have to be agreed by the research team and approved by the Ethical Committee for Health of the Faculty of Sport Sciences and Physical Education of the University of Coimbra before application. Written informed consent will be obtained from the participants after the study has been completely explained (i.e., study procedures, objectives, potential risks, and expected results). All participants will receive a signed copy of the consent form. Each participant will receive a unique coded identification (ID) number to maintain their confidentiality, and all experimental data will be recorded using these ID codes. The files that connect participant ID codes to other identifying information (like informed consent forms, lists, appointment books) will be stored in a separate locked file and only the PI will have access [46]. Moreover, all databases inherent to this research will be ensured with password-protected access systems [46]. Participants will be given an estimate of when the study will end. The results of this study will be presented and published regardless of the magnitude or direction of effect, at the planned target of 3 to 6 months after the end date of the intervention (or at an earlier date if the circumstances allow). Communication of the results to the public will start with the study participants, their families, and colleagues. Communication to the scientific community will be performed by participating in national/international conferences/congresses. Authorship of the manuscripts resulting from this research will be based on the following criteria: significant contributions to the conceptualization or design of the research project, formal analysis and/or interpretations of the data, and drafting and/or revising and editing the manuscript critically.

## Supporting information

S1 Checklist Completed SPIRIT checklist. (DOC)
